# 2200 The effect of antipyretics and fever on the mortality of mechanically ventilated patients

**DOI:** 10.1017/cts.2018.131

**Published:** 2018-11-21

**Authors:** Emily M. Evans, Rebecca J. Doctor, Brian M. Fuller, Richard S. Hotchkiss, Anne M. Drewry

**Affiliations:** 1 Institute of Clinical and Translational Sciences, Washington University in St. Louis; 2 Washington University; 3 Washington University School of Medicine

## Abstract

OBJECTIVES/SPECIFIC AIMS: (1) To evaluate clinical outcomes in mechanically ventilated patients with and without fever. We hypothesize that, after adjusting for confounding factors such as age and severity of illness: (a) In septic patients, fever will be associated with improved clinical outcomes. (b) In nonseptic patients, fever will be associated with worse clinical outcomes. (2) To examine the relationship between antipyretics and mortality in mechanically ventilated patients at risk for an acute lung injury. We hypothesize that antipyretics will have no effect on clinical outcomes in mechanically ventilated patients with and without sepsis. METHODS/STUDY POPULATION: This is a retrospective study of a “before and after” observational cohort of 1705 patients with acute initiation of mechanical ventilation in the Emergency Department from September 2009 to March 2016. Data were collected retrospectively on the first 72 hours of temperature and antipyretic medication from the EHR. Temperatures measurements were adjusted based on route of measurement. Patients intubated for cardiac arrest or brain injury were excluded from our primary analysis due to the known damage of hyperthermia in these subsets. Cox proportional hazard models and multivariable linear regression analyzed time-to-event and continuous outcomes, respectively. Predetermined patient demographics were entered into each multivariable model using backward and forward stepwise regression. Models were assessed for collinearity and residual plots were used to assure each model met assumptions. RESULTS/ANTICIPATED RESULTS: Antipyretic administration is currently undergoing analysis. Initial temperature results are reported here. In the overall group, presence of hypothermia or fever within 72 hours of intubation compared with normothermia conferred a hazard ratio (HR) of 1.95 (95% CI: 1.48–2.56) and 1.31 (95% CI: 0.97–1.78), respectively. Presence of hypothermia and fever reduced hospital free days by 3.29 (95% CI: 2.15–4.42) and 2.34 (95% CI: 1.21–3.46), respectively. In our subgroup analysis of patients with sepsis, HR for 28-day mortality 2.57 (95% CI: 1.68–3.93) for hypothermia. Fever had no effect on mortality (HR 1.11, 95% CI: 0.694–1.76). Both hypothermia and fever reduced hospital free days by 5.39 (95% CI: 4.33–7.54) and 3.98 (95% CI: 2.46–5.32) days, respectively. DISCUSSION/SIGNIFICANCE OF IMPACT: As expected, both hypothermia and fever increased 28-day mortality and decreased hospital free days. In our sepsis subgroup, hypothermia again resulted in higher mortality and fewer hospital free days, while fever did not have a survival benefit or cost, but reduced hospital free days. Antipyretic administration complicates these findings, as medication may mask fever or exert an effect on survival. Fever may also affect mechanically ventilated septic patients differently than septic patients not on mechanical ventilation. Continued analysis of this data including antipyretic administration, ventilator free days and progression to ARDS will address these questions.

